# Multi-Omics Analysis Revealed a Significant Alteration of Critical Metabolic Pathways Due to Sorafenib-Resistance in Hep3B Cell Lines

**DOI:** 10.3390/ijms231911975

**Published:** 2022-10-09

**Authors:** Kholoud Y. I. Abushawish, Sameh S. M. Soliman, Alexander D. Giddey, Hamza M. Al-Hroub, Muath Mousa, Karem H. Alzoubi, Waseem El-Huneidi, Eman Abu-Gharbieh, Hany A. Omar, Sara M. Elgendy, Yasser Bustanji, Nelson C. Soares, Mohammad H. Semreen

**Affiliations:** 1 College of Pharmacy, University of Sharjah, Sharjah P.O. Box 27272, United Arab Emirates; 2 Research Institute of Medical and Health Sciences, University of Sharjah, Sharjah P.O. Box 27272, United Arab Emirates; 3 Research Institute of Science and Engineering, University of Sharjah, Sharjah P.O. Box 27272, United Arab Emirates; 4 College of Medicine, University of Sharjah, Sharjah P.O. Box 27272, United Arab Emirates; 5 School of Pharmacy, The University of Jordan, Amman 11942, Jordan

**Keywords:** proteomics, metabolomics, parental Hep3B cells, sorafenib-resistant Hep3B cells, UHPLC-QTOF-MS

## Abstract

Hepatocellular carcinoma (HCC) is the second prominent cause of cancer-associated death worldwide. Usually, HCC is diagnosed in advanced stages, wherein sorafenib, a multiple target tyrosine kinase inhibitor, is used as the first line of treatment. Unfortunately, resistance to sorafenib is usually encountered within six months of treatment. Therefore, there is a critical need to identify the underlying reasons for drug resistance. In the present study, we investigated the proteomic and metabolomics alterations accompanying sorafenib resistance in hepatocellular carcinoma Hep3B cells by employing ultra-high-performance liquid chromatography quadrupole time of flight mass spectrometry (UHPLC-QTOF-MS). The Bruker Human Metabolome Database (HMDB) library was used to identify the differentially abundant metabolites through MetaboScape 4.0 software (Bruker). For protein annotation and identification, the Uniprot proteome for Homo sapiens (Human) database was utilized through MaxQuant. The results revealed that 27 metabolites and 18 proteins were significantly dysregulated due to sorafenib resistance in Hep3B cells compared to the parental phenotype. D-alanine, L-proline, o-tyrosine, succinic acid and phosphatidylcholine (PC, 16:0/16:0) were among the significantly altered metabolites. Ubiquitin carboxyl-terminal hydrolase isozyme L1, mitochondrial superoxide dismutase, UDP-glucose-6-dehydrogenase, sorbitol dehydrogenase and calpain small subunit 1 were among the significantly altered proteins. The findings revealed that resistant Hep3B cells demonstrated significant alterations in amino acid and nucleotide metabolic pathways, energy production pathways and other pathways related to cancer aggressiveness, such as migration, proliferation and drug-resistance. Joint pathway enrichment analysis unveiled unique pathways, including the antifolate resistance pathway and other important pathways that maintain cancer cells’ survival, growth, and proliferation. Collectively, the results identified potential biomarkers for sorafenib-resistant HCC and gave insights into their role in chemotherapeutic drug resistance, cancer initiation, progression and aggressiveness, which may contribute to better prognosis and chemotherapeutic outcomes.

## 1. Introduction

Hepatocellular carcinoma (HCC) is one of the most commonly diagnosed cancer types and has a low two-year survival rate (50%) [1]. It is known to develop drug resistance and is hard to be diagnosed in its early stages. It accounts for the second prominent cause of cancer-related death worldwide [2]. HCC occurs typically as a progression of liver diseases related to numerous contributing factors, including toxins such as aflatoxin, alcohols, viral liver diseases such as chronic hepatitis B virus (HBV) and C virus (HCV), autoimmune and other metabolic-based diseases such as diabetes, hemochromatosis, and non-alcoholic fatty liver disease [3,4,5]. Hepatic fibrosis triggered by chronic HBV or HCV viral infection is known to be the primary predisposing factor in HCC development [6].

Early detection methodologies for HCC are often ineffectual. The diagnostic biomarker α-fetoprotein (AFP) has been the main biomarker for HCC in the last 40 years. Nevertheless, it is associated with shortcomings of low sensitivity and specificity and remains unsatisfactory [7,8]. Most HCC patients are diagnosed in the advanced stages, in which several treatment modalities are followed, including the tyrosine kinase inhibitor targeted therapy (sorafenib), systematic chemotherapy using cytotoxic agents (cisplatin and 5-fluorouracil), trans-arterial chemoembolisation, external irradiation, liver resection and orthotopic liver transplantation (OLT). Despite the variety of treatment protocols, only OLT and surgical resection are curative [9,10]. Sorafenib, a multiple-target tyrosine kinase inhibitor, is utilized as the first-line therapy for patients with advanced HCC stage. However, it is associated with high costs that have been identified as a pivotal clinical obstacle, in addition to common adverse events presented in HCC patients, including hypertension, bleeding, cardiac ischemia, rash, and diarrhea [11]. Although around 30% of HCC patients can benefit from sorafenib treatment, many unfortunately acquire drug resistance within a six-month treatment period [12,13,14,15]. As a result, it is critical to identify the underlying causes of sorafenib-resistant HCC so that a novel treatment strategy can be proposed to overcome drug resistance.

Cancer drug resistance is a complicated process that can arise from various alterations in cell cycle and DNA repair, cell apoptosis and autophagy, drug efflux, cancer stem cells (CSCs) generation, abnormal activation of signaling pathways, epithelial-mesenchymal transition (EMT) induction, mutations of drug targets and increase in drug metabolism [16]. Numerous mechanisms involved in the resistance to sorafenib including regulated cell death, phosphatidylinositol 3-kinase (PI3K)/Akt pathway, JAK-STAT pathways, immune microenvironment, EMT, activation of hypoxia-inducible pathways, microRNAs (miRNAs) and epigenetic regulation [13,17]. Despite our knowledge of drug resistance in cancer, the latest and innovative methodologies are important to capture those biomarkers responsible for this resistance. Omics technologies (proteomics, metabolomics, etc.) have recently displayed rapid progress in the early diagnosis of diseases and appraising the nature of disease mechanisms and pathogenesis [7,18]. High-throughput proteomic and metabolomic technologies offer an effective tool for the identification of distinguishing biomarkers of tumorigenesis, as well as the evaluation of therapeutic efficacy, drug resistance and better knowledge of the biological pathways involved [19]. 

Mass spectrometry (MS)-based techniques combined with separation techniques, liquid chromatography (LC) and gas chromatography (GC) have been at the forefront in biomarker innovation studies [20]. The LC-MS/MS technique provides superior sensitivity and precision in quantifying metabolites and proteins. This is due to the identification of the *m*/*z* ratio of the precursor ions via two successive MS detections [21]. 

This research study was performed using UHPLC-QTOF-MS, a novel platform of mass spectrometry technology with a high level of identification and profiling of metabolites and proteins in complex samples. Although previous studies had revealed metabolic and proteomic changes in Hep3B cells [22,23,24] there is no metabolomics and proteomics study to date that has comparatively profiled the metabolomic and the proteomic composition of parental and resistant Hep3B cell lines via UHPLC-QTOF-MS analysis to study drug resistance.

The data obtained complements earlier studies describing the proteomic and metabolomic profiles of parental and sorafenib-resistant Hep3B HCC and, therefore, provide new insights into the potential drug-resistance targets for studying the underlying mechanisms in hepatic carcinoma.

## 2. Results

### 2.1. Sorafenib-Resistant Hep3B Cells

**Figure 1:**
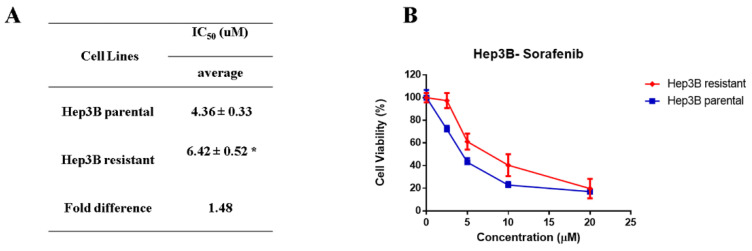
The generated Hep3B resistant subclones are less sensitive to sorafenib (**A**) Table representation of the IC50 of sorafenib in parental and resistant Hep3B cells after 48 h treatment. IC50 are means ± SD of three individual experiments (*n* = 3), * (*p* value < 0.05). (**B**) Concentration-response curves of sorafenib parental Hep3B cells and the resistant cells. MTT assay was conducted at 48 h after treatment. Points, mean; bars, SD (*n* = 3).

MTT cell viability assay was conducted to assess the sensitivity of parental and resistant Hep3B cells to sorafenib. The results revealed that Hep3B resistant cells were significantly (*p* value < 0.05) less sensitive to sorafenib by ~1.5 fold compared to parental cells (Figure 1). The IC50 of sorafenib in Hep3B resistant cells and parental cells were 6.42 ± 0.52 and 4.36 ± 0.33 µM, respectively (Figure 1). The results indicated that Hep3B cells acquired resistance to sorafenib. 

### 2.2. Metabolomics Analysis Revealed a Significant Change/Shift of Metabolic Pathways Due to Sorafenib Resistance

**Figure 2:**
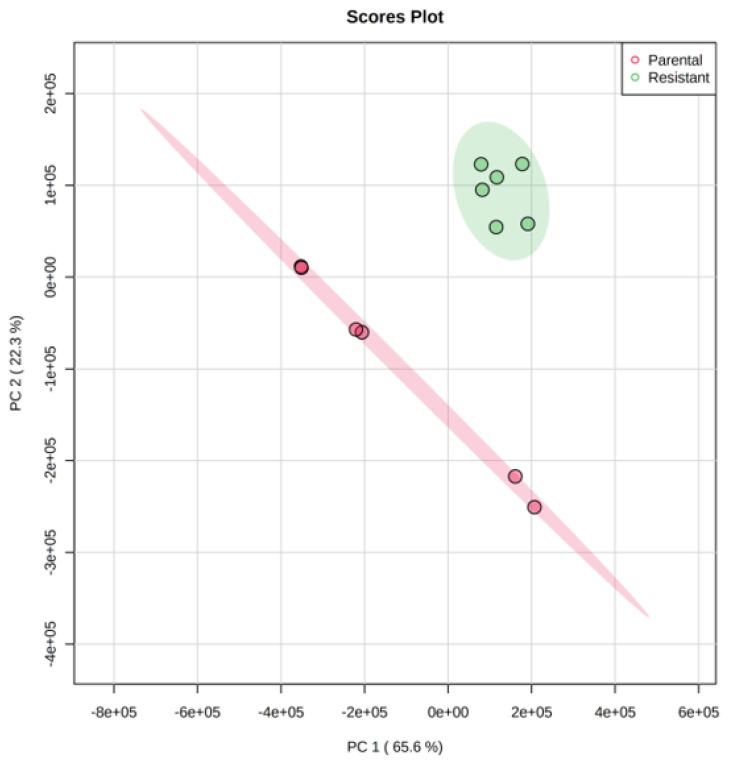
PCA of metabolic profile of parental and resistant Hep3B cells.

**Figure 3:**
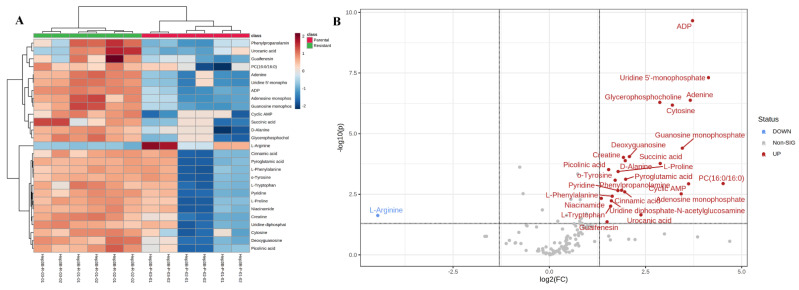
(**A**) Hierarchical cluster analysis and heatmap analysis of differentially abundant metabolites in resistant and parental phenotypes of Hep3B cells. Heatmap showing the abundance levels and clustering of the significantly differentially deregulated metabolites (two-sample *t*-test with *p* value < 0.05; log2FC < 2; resistant/parental Hep3B cells). Metabolite’s intensities up-regulated are colored in red while down-regulated are colored in blue. Row headings represent metabolites names and column headings represent samples. Euclidean distance measure and Ward linkage analysis were used to carry out hierarchical clustering using the metabolomics data. Heatmap analysis showed that metabolites clustered into two separate groups, a group representing the resistant Hep3B cells and the other group representing the parental Hep3B cells. (**B**) Volcano plots showing the metabolites that altered significantly in resistant Hep3B cells vs. parental Hep3B cells. Scatter plot showing the metabolites log2 fold-change (resistant/parental) plotted against the -log10(*p* value) highlighting the differentially metabolites (two-sample *t*-test with BH FDR < 0.05). Differentially significant metabolites which are increased in resistant Hep3B cells are indicated in red. Differentially significant metabolites which are decreased in resistant Hep3B cells are indicated in blue. Non-significant metabolites are indicated in grey.

Metabolomics analysis of sorafenib-resistant Hep3B versus parental cells revealed 134 metabolites separated into two clusters, each representing a cell type (Figure 2). Sparse partial least squares-discriminant analysis (sPLS-DA) showed no overlap between the groups, indicative of significant differences between both clusters (Supplementary material: Figure S1). Utilizing a two-tailed independent students *t*-test, a potential variation in the metabolic profiles of parental and resistant Hep3B cells was detected. An overall number of 27 metabolites were changed significantly (*p* value < 0.05) with log2-fold change < 2. Hierarchical clustering demonstrated a clear separation of the metabolites between resistant Hep3B and parental cells (Figure 3A). L-arginine was the only metabolite whose abundance decreased in resistant Hep3B cells in comparison to parental Hep3B cells. On the other hand, 26 metabolites increased in resistant Hep3B cells. These included adenosine monophosphate (AMP), adenosine di-phosphate (ADP), creatine, pyroglutamic acid and succinic acid (Figure 3B and Table 1), all of which are important for energy production pathways [25,26,27,28,29]. In addition, deoxyguanosine, uridine 5′-monophosphate, guanosine monophosphate, adenine and cytosine might support the de novo synthesis of nucleotides and have been found to be elevated in resistant Hep3B cells (Figure 3B and Table 1) [30]. Moreover, other metabolites like D-alanine, L-proline and o-tyrosine increased in resistant Hep3B cells (Figure 3B and Table 1), might be involved in some dysregulated metabolic pathways in cancer cells [31]. Furthermore, some elevated metabolites in resistant Hep3B cells may contribute to choline metabolism included in glycerophosphocholine and phosohatidylcholine (PC) (16:0/16:0) [32]. Other important metabolites play a vital role in transcription, proliferation and apoptosis, such as cyclic AMP and uridine diphosphate-N-acetylglucosamine, which were elevated in resistance Hep3B cells (Figure 3B and Table 1) [33,34,35].

**Figure 4:**
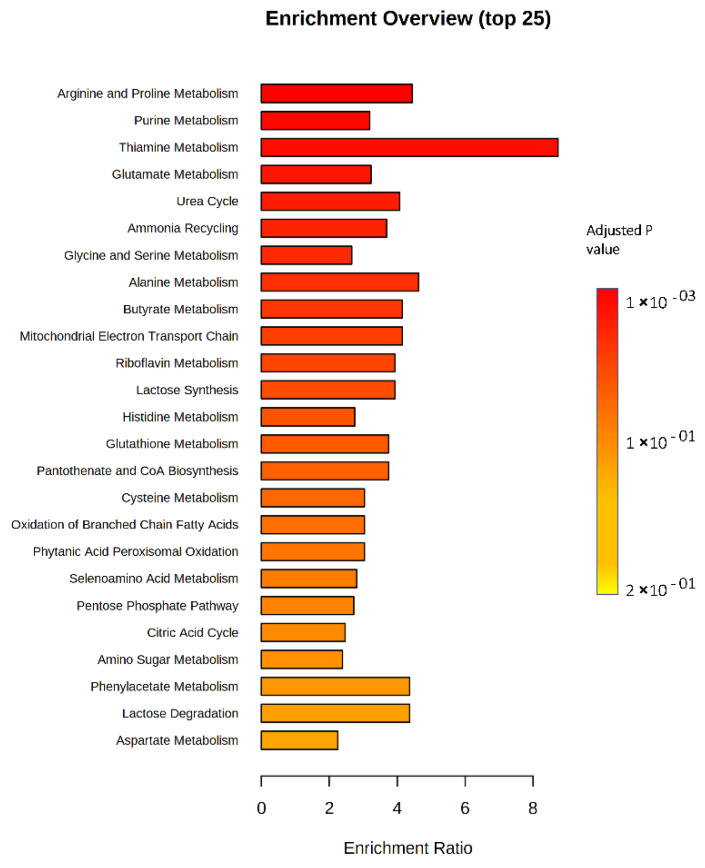
Metabolomic set functional analysis showing the most altered functional metabolic pathways in resistant Hep3B cells. The graph was obtained using pathway enrichment analysis in the MetaboAnalyst software through plotting the *p* values on the y-axis.

Enriched metabolic pathway analysis revealed a significant impact on nucleotide metabolism, including purine metabolism and the energy-producing pathways, specifically the mitochondrial electron transport chain (ETC) and butyrate metabolism. Furthermore, important biochemical processes such as the urea cycle, ammonia recycling and amino acid metabolisms like proline, glutamate, glycine, alanine and serine were significantly impacted (Figure 4).

### 2.3. Proteomics Analysis Indicate a Unique Protein Profile Associated with Hep3 Drug Resistance

**Figure 5:**
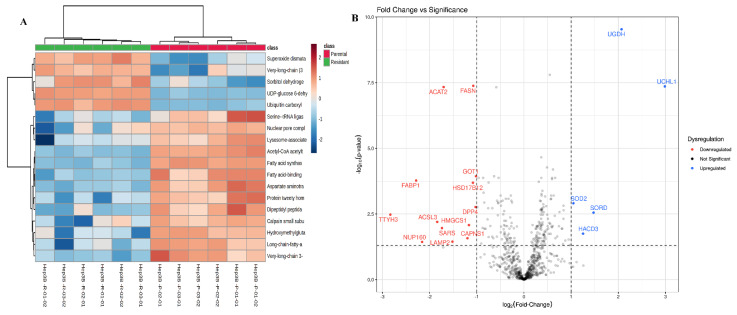
(**A**) Hierarchical cluster analysis and heatmap of differentially abundant proteins in resistant and parental phenotypes of Hep3B cells. Heatmap showing the abundance levels and clustering of the significantly differentially deregulated proteins (two-sample *t*-test with adjusted *p* value < 0.05; log2FC < 1; resistant/parental Hep3B cells). High protein intensities are colored red while low intensities are colored blue. Row headings represent protein names and column headings represent samples. Euclidean distance measure and Ward linkage analysis were used to carry out hierarchical clustering using the differentially abundant protein data. Heatmap analysis showed that the samples clustered into two separate groups, a group representing the resistant Hep3B cells and the other group representing the parental Hep3B cells. (**B**) Volcano plot showing those proteins which were altered significantly in resistant Hep3B cells vs. parental phenotype. Scatter plot showing the proteins log2-transformed fold-change (resistant/parental) plotted against the -log10(*p* value) highlighting the differentially proteins (two-sample *t*-test with BH FDR < 0.05). Down-regulated and up-regulated proteins are indicated in red and blue, respectively.

In our proteomics analysis, 168,886 spectra were identified, and the redundant peptides were filtered out, yielding 7809 non-redundant peptides, of which 7439 were unique to their respective protein groups. The false discovery rate (FDR) for peptide identifications was 0.29%, while it was found to be 0.92% for protein identifications. A total of 1167 proteins were confidently detected and identified in parental and resistant Hep3B cells. After filtration of the data with Perseus software, proteins with 70% valid values kept yielding 730 proteins. Proteomics analysis revealed 730 proteins; of them, 212 proteins were significant (*p* value < 0.05) and from these, 18 proteins were significantly (*p* value < 0.05) changed between parental and resistant Hep3B cells with log2(fold-change) >1. Hierarchical clustering revealed a clear clustering of the proteins’ expression in resistant Hep3B cells compared to parental phenotype (Figure 5A). A total of 13 proteins significantly decreased in abundance in the resistant cells compared to parental cells, which demonstrated different roles linked to cancer (Figure 5B, Table 2 and Supplementary material: Summary Table S1). Some of these proteins are vital for cancer cell survival and migration. Proteins such as calpain small subunit 1 play a role in regulating cell proliferation, whereas dipeptidyl peptidase 4 participates in cell adhesion and migration (Figure 5B, Table 2 and Supplementary material: Summary Table S1). In addition, other decreased proteins in the resistant Hep3B cells are important in biosynthetic processes, metabolism, signaling and transporting in cancer. These include very-long-chain 3-oxoacyl-CoA reductase proteins, which contribute to the very-long-chain fatty acid biosynthetic process, as well as hydroxymethylglutaryl-CoA synthase, cytosolic fatty acid CoA ligase Acsl3 and cytoplasmic Acetyl-CoA acetyltransferase, all of which play a crucial role in cancer cell metabolism. Moreover, cytoplasmic aspartate aminotransferase participates in Notch signaling pathway, while protein tweety homolog 3 is involved in chloride transport and ion transmembrane transport (Figure 5B, Table 2 and Supplementary material: Summary Table S1). Furthermore, hepatic fatty acid binding proteins are involved in apoptosis and help regulate the cell death process. They have also shown lower abundance levels in resistant Hep3B cells (Figure 5B, Table 2 and Supplementary material: Summary Table S1). Additionally, some proteins participate in the hemostasis and cancer cell survival: lysosome-associated membrane glycoprotein 2, which is crucial for chaperon mediated-autophagy; fatty acid synthase, which is important in cellular immune responses; serine-tRNA ligase, which is involved in transcription regulation; and cytoplasmic nuclear pore complex protein Nup160, nucleocytoplasmic and protein transport, which were found to decrease in resistant Hep3B cells (Figure 5B, Table 2 and Supplementary material: Summary Table S1). 

The proteins that increased in resistant Hep3B cells are as follows: ubiquitin carboxyl-terminal hydrolase isozyme L1, which is a protein that contributes to cellular response to xenobiotic stimulus, cell population and proliferation; mitochondrial superoxide dismutase, which plays a crucial role in intrinsic apoptotic signaling pathway in response to oxidative stress; sorbitol dehydrogenase, which is an enzyme responsible for sorbitol catabolic process; UDP-glucose 6-dehydrogenase, which is involved in the metabolic process; and very-long-chain (3R)-3-hydroxyacyl-CoA dehydratase 3, which participates in the very long-chain fatty acid biosynthetic process (Figure 5B, Table 2 and Supplementary material: Summary Table S2). 

**Figure 6:**
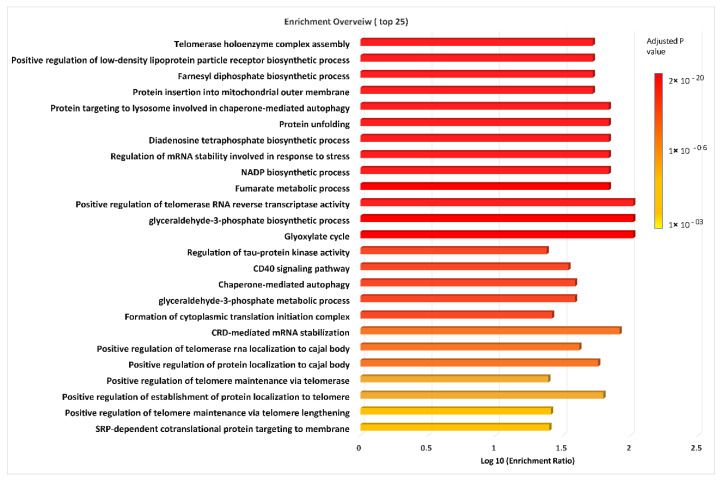
Visualization of enrichment analysis for gene ontology biological process (GOBP) terms using String software version 11.5. The graph shows top 25 significant GOBP terms colored according to their respective enrichment *p* values.

To identify the gene ontology biological process (GOBP) terms associated with dysregulated proteins, we used gene ontology enrichment analysis (GOEA) using String 11.5. From among the top significantly enriched pathways, some pathways have been found to have impact in cancer development, survival, cellular homeostasis, migration and progression. These include telomerase holoenzyme complex assembly, positive regulation of telomerase RNA reverse transcriptase activity [36], positive regulation of low-density lipoprotein particle receptor biosynthetic process [37], farnesyl diphosphate biosynthetic process [38], protein insertion into mitochondrial outer membrane [39], protein targeting to lysosome involved in chaperone-mediated autophagy [40], protein unfolding [41], diadenosine tetraphosphate biosynthetic process [42], regulation of mRNA stability involved in response to stress [43], the NADP biosynthetic process [44], the fumarate metabolic process [45] and the glyceraldehyde-3-phosphate biosynthetic process [46] (Figure 6). Our findings indicate that a number of differentially abundant protein are related to energy production, anabolic processes, drug resistance and cancer cell survival and growth in sorafenib resistance (Supplementary material: Summary Tables S1 and S2).

### 2.4. Multi-Omics Integrated Analysis Demonstrated the Involvement of Major Pathways in HCC Development and Drug Resistance

**Figure 7:**
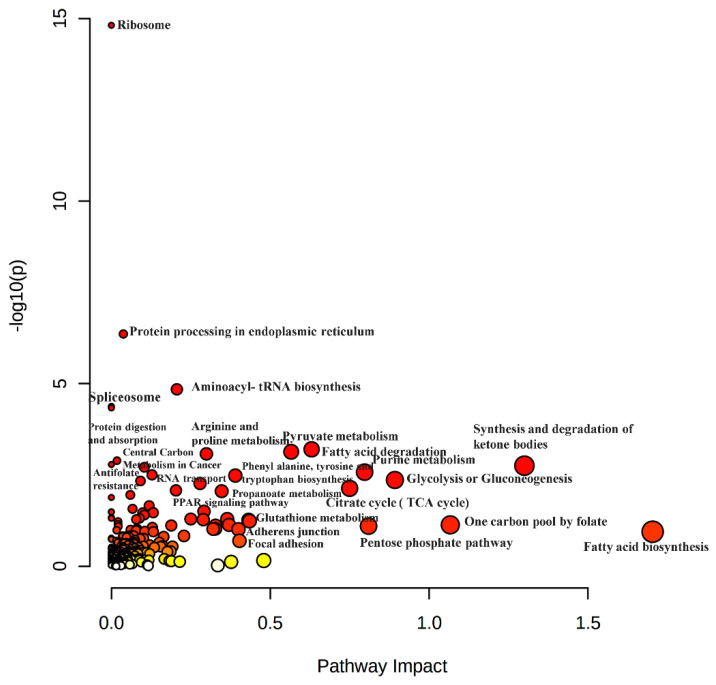
Visualization of joint pathway enrichment analyses. Graph that resulted from the joint pathway enrichment analysis using MetaboAnalyst of the proteins and metabolites that altered significantly (*p* value < 0.05) in resistant Hep3B cells vs. parental Hep3Bcells. Nodes are colored according to *p* value. Significant pathways are colored red, while non-significant pathways are colored yellow to white. Nodes are sized according to the number of associated members proteins and/or metabolites.

A joint pathways analysis was performed using MetaboAnalyst to generate an integrative analysis that merges the metabolomics and proteomics profiles. All 212 proteins and 39 metabolites that were significantly altered (*p* value < 0.05) in resistant and parental Hep3B cells were included. Several pathways were highly impacted, including the metabolic pathways for several amino acids such as arginine, glutamate, proline, glycine, alanine and serine. Additionally, pathways related to the protein synthesis including in the ribosome, which are proteins in the endoplasmic reticulum and aminoacyl-tRNA biosynthesis, were observed to be dysregulated. Other enriched pathways important for energy production and anabolic and catabolic pathways included those involving the peroxisome, fatty acid degradation, pyruvate metabolism, central carbon metabolism in cancer, protein digestion and absorption, synthesis and degradation of ketone bodies, glycolysis or gluconeogenesis, citrate and the TCA cycle. In addition to these pathways, other pathways were found to be important for transcription and RNA transporting, such as the PPAR signaling pathway, spliceosome and RNA transport. Interestingly, antifolate resistance was one of the top 25 enriched pathways (Figure 7). 

## 3. Discussion

In this study, a multi-omics (metabolomics and proteomics)-based comparative analysis of sorafenib-resistant Hep3B cells compared to parental cells was performed. The metabolites’ and proteins’ patterns of expression in resistant Hep3B cells were differentially abundant in comparison to their corresponding parental cells (Figure 3B and Figure 5B). The difference developed due to drug resistance is associated with alteration in several biological mechanisms [16]. 

### 3.1. Comparative Metabolomics Revealed That Sorafenib-Resistant Hep3B Cells Demonstrated a Significant Dysregulation in Amino Acid and Nucleotide Metabolic Pathways, Energy Production Pathways and Other Cancer Aggression, Migration, Proliferation and Drug Resistance-Related Pathways

Our metabolomic findings showed significant variations in the levels of some amino acids in the resistant compared to the parental Hep3B cells, including D-alanine, L-proline and o-tyrosine (Figure 3B), and, interestingly, most of the significantly impacted pathways in metabolomic enrichment analysis are amino acid metabolic pathways such as proline, glutamate, alanine, serine and glycine metabolic pathways (Figure 4). These metabolic pathways are well-known to be dysregulated in cancer cells, since cancer has the capability to rewire the amino acid metabolism to support their survival and chemoresistance through protein anabolism, de novo nucleotide metabolism, energy generation, epigenetic modulation and maintenance of cellular redox homoeostasis [31,47]. In addition, the energy-producing pathways were highly impacted, specifically the mitochondrial electron transport chain and butyrate metabolism (Figure 4). This is in line with the superior demand of tumor cells for elevated levels of ATP to support their various metabolic pathways, survival, proliferation and drug insensitivity [48,49,50,51,52]. In this context, one recent article demonstrated that multidrug resistance of melanoma relies on oxidative ATP production to support the rewired metabolic pathways. Mitochondrial ETC inhibition by biguanides and antidiabetics increase the chemotherapeutic effect [52]. Besides amino acid and energy metabolism, purine metabolism was found to be significantly changed in resistant Hep3B cells, which might be due to the reprogramming of the nucleotide metabolism to maintain resistance to chemotherapeutics [30,53,54].

Targeting such upregulated amino acid metabolic pathways might shine a spotlight on a potential therapeutic target in resistant cancer cells. Consistent with our observation, a recent publication showed that targeting overexpressed glutamine metabolism in sorafenib-resistant HCC through the inhibition of peroxisome proliferator-activated receptor-δ (PPARδ) enabled the overcoming of sorafenib resistance in HCC [55]. To the best of our knowledge, our study is the first to demonstrate the inclusion of D-alanine and o-tyrosine in sorafenib-resistance in Hep3B cells. Thus, we hypothesized that these two amino acids could be valuable biomarkers in drug resistance in HCC.

Resistant Hep3B cells showed a significant increase in the level of phosphatidylcholine (PC 16:0/16:0), a form of glycerophospholipids, which is a component of cellular membranes that participates in numerous cellular functions (Figure 3B). It has been reported that elevated levels of (PC 16:0/16:0) can participate with other phospholipids in discriminating HCC patients from cirrhotic control patients [56]. Their importance arises from the fatty acyl residues that greatly impact cancer cells that differentiate from drug-resistant and drug-sensitive cells. It has been previously reported that phosphatidylcholine synthesis in colorectal cancer is linked with the expression of lysophosphatidylcholine acyltransferase 2 (LPCAT2) in the lipid droplet (LD) accumulation of cancer cells. LD accumulation was shown to overcome the therapeutic effect of oxaliplatin and 5-fluorouracil by curtailing the endoplasmic reticulum (ER) stress responses and impairing the activation of the caspase cascade [57]. Furthermore, PC (16:0/16:0) was found to accumulate in the lymphoid, suggesting its engagement in the inflammatory reactions of cancer cells [58]. In this context, we assumed that PC (16:0/16:0) could be a valuable potential biomarker for resistant HCC.

Excitingly, one of the significantly increased metabolites in resistant Hep3B cells was succinic acid (Figure 3B). Succinic acid is an intermediate metabolite in the TCA cycle in mitochondria that plays a vital role in many metabolic pathways, especially ATP production [27]. It has been reported that succinic acid is accumulated as a result of a mutation in the succinate dehydrogenase (SDH) enzyme, which is required in the conversion of succinate to fumarate in mitochondria. Moreover, it is the main driver of tumorigenesis in hereditary paragangliomatosis with phaeochromocytomas (HPGL) [59]. This is most likely due to the activation of the hypoxia-inducible factor (HIF) pathway and, consequently, an angiogenic increase in an anaerobic metabolism called “pseudo-hypoxic drive” [60,61,62]. Remarkably, this might enable resistance to certain apoptotic signals and could be a biomarker for drug insensitivity.

Our metabolomic results also revealed that a group of metabolites, including uridine 5’-monophosphate, guanosine monophosphate, adenine, cytosine and deoxyguanosine, all of which are involved in the synthesis of nucleotides, are significantly increased in resistant Hep3B cells (Figure 3B). All are correlated with the significantly enriched purine metabolism (Figure 4). It is worth stating that the survival of cancer cells requires an adequate supply of nucleotide pools to maintain increased DNA, RNA and protein synthesis, energy production and conservation, cancer cell growth and proliferation via upregulated de novo nucleotide metabolism [30]. 

Metabolic enrichment analysis revealed significant dysregulation of important biological processes in the resistant Hep3B cells, such as urea cycle (UC), ammonia recycling and arginine metabolism. These may be associated with the significantly decreased abundance of L-arginine that was identified in our metabolomics results (Figure 3B and Figure 4). It has been stated that UC and ammonia recycling play an important role in liver cancer, in which the cancerous cells might release ammonia into the blood circulation [63] which is in turn metabolized to urea through the UC, which is then secreted into the blood circulation [64]. Thus, circulating urea may be considered a biomarker for HCC [65]. Interestingly, dysregulation of UC in cancer is linked with the reduced generation of nitrogen waste and augmented re-routing of carbon and nitrogen to anabolic pathways due to the alteration in the expression of the UC [66]. One recently published article reported that decreased argininosuccinate synthetase1 (ASS1) expression, an important enzyme in UC, in doxorubicin-resistant sarcoma might greatly impact drug resistance [67]. Additionally, ASS1 is one of the rate-limiting enzymes in the de novo arginine metabolism, and it has been reported that hepatocellular cells do not express ASS1 [68,69]. We speculated that dysregulation of UC and arginine metabolism, along with a decrease of L-arginine in resistant Hep3B cells, might be due to the lower expression of argininosuccinate synthetase1 (ASS1), which has been linked to drug resistance. Consequently, decreased L-arginine levels could be a beneficial biomarker for drug resistance in HCC.

### 3.2. Comparative Proteomics Indicated That Resistant Hep3B Cells Significantly Alter Specific Pathways That Have a Great Impact on the Development of Chemotherapeutic Resistance

We identified proteins associated with sorafenib resistance in Hep3B cells and its relationship to cancer aggressiveness, migration and proliferation. Two important proteins were identified in this context: ubiquitin carboxyl-terminal hydrolase isozyme L1 (UCHL1) and mitochondrial superoxide dismutase. Both proteins significantly increased in resistant Hep3B cells compared to parental phenotype (Figure 5B). UCHL1 is a neuronal-related protein that scavenges ubiquitin at its C-terminal peptide, isopeptide, amide, ester and thioester bonds through thiol-related hydrolysis [70]. Despite its neuronal expression, various studies have proven its overexpression in different cancer types [71,72,73]. UCHL1 plays a vital role in anti-apoptosis, cancer cell invasion, migration and proliferation. Moreover, it enhances the multi-drug resistance in different cancer cells and tissues [73,74,75]. A previous study showed that UCHL1 overexpression supported apoptosis in verapamil- and Adriamycin-treated hepatoma cell lines (BEL-7402 and SMMC-7721) [76]. Verapamil reverses the multi-drug resistance (MDR) through competition on P-glycoprotein chemotherapeutic associated binding sites and increases chemotherapeutic drug retention in cancer cells [77]. Knockdown of UCHL1 by siRNA attenuated the cell apoptosis effect of combined drugs, which indicated the important role of UCHL1 in the verapamil-mediated reversal of drug resistance [76]. In another study, UCHL1 overexpression improves the early diagnosis and detection rate and provides targeted drug therapy for lung adenocarcinoma [75]. The underlying mechanism of UCHL1 mediated MDR to P-glycoprotein (P-gp) substrate drugs and the increased invasion and migration in resistant breast cancer cells compared to the sensitive phenotype, which might be associated with MAPK/Erk pathway activation [74]. This resulted in the upregulation of MDR1 gene, metalloproteinase inducer (CD147) and matrix metalloproteinases (MMP2 and MMP9), which are highly associated with the cancer cells invasiveness and migration, and MDR in cancer [74]. Additionally, increased UCH-L1 levels in advanced breast cancer patients who did not respond to chemotherapy led to overexpression of epidermal growth factor receptor (EGFR) and an increase in cancer cell invasion, metastasis and shortened the rate of survival [78]. Furthermore. it has been demonstrated that p-ERK, EGFR and p-Akt [79] expressions were upregulated in the sorafenib-resistant cells. The resistance here triggered by overexpression of EGFR that might led to Akt and ERK signaling pathway activation and enhancing the proliferation and survival of HCC cells [80]. In this context, as mentioned above, UCHL1 is associated with MAPK/Erk pathway activation and overexpression of EGFR, which might have a great impact on sorafenib resistance in HCC. To the best of our knowledge, our study was the first to reveal the elevated levels of UCHL1 in resistant Hep3B cells. Our findings with the support of the published data suggested that UCH-L1 might be a potential prognostic biomarker and valuable target in chemoresistance, and its target may improve the chemotherapeutic outcomes in HCC.

The mitochondrial superoxide dismutase (SOD2) was also upregulated in resistant Hep3B cells compared to the corresponding parental phenotype (Figure 5B). SOD2 is a mitochondrial enzyme responsible for the conversion of superoxide to hydrogen peroxide and in the protection against reactive oxygen species (ROS)-mediated cell death [81]. Many cancers have high levels of expression of SOD2, particularly in the metastatic and aggressive phenotypes [82,83]. Additionally, SOD2 plays a chief role in the clearance of chemotherapeutics-induced ROS. Overexpression of miR-146a, a micro-RNA dysregulated in many cancers, in epithelial ovarian cancer (EOC) downregulates SOD2 expression considerably, which consequently elevates ROS levels and leads to suppression of cell proliferation, promotion of apoptosis and enhancement of chemosensitivity [84]. Further study confirmed that SOD2 overexpression is a biomarker in predicting poorer prognosis with endometrioid ovarian carcinoma and shows extreme resistance to chemotherapeutics-mediated oxidative stress [85]. The recent publication also demonstrated the anticancer effect of fraxetin, an extract from Fraxinus rhynchophylla, on Hep3B and Huh7 cells by lowering the SOD2 expression and disrupting antioxidant mechanisms in the mitochondria [86]. Consequently, we hypothesized that SOD2 overexpression in resistant Hep3B cells could be a potential biomarker for drug resistance, as well as critical in improving the effectiveness of the chemotherapeutic.

Sorbitol dehydrogenase (SORD) is a crucial enzyme in the polyol pathway that converts sorbitol to fructose. It was also identified in our proteomics analysis to be significantly increased in abundance in resistant cells (Figure 5B). Polyol pathway promotes the cancer cell progression and aggressiveness through sugar alcohols produced by two enzymes: SORD and aldo-keto reductase [87]. More specifically, the high level of fructose produced via SORD in pancreatic cancers was proven to participate in cancer cell proliferation via nucleic acids biosynthesized by the induction of thiamine-dependent transketolase flux and the metabolic nonoxidative pentose phosphate pathway (PPP) [88]. It has been reported that the levels of sugar alcohol in the blood increase from the early to late stages of HCC patients [89]. It has been suggested that the prognostic ability of SORD along with AFP for recurrence-free survival (RFS) after surgical resection (SR) should be incorporated in HCC patients’ management [90]. Elevated SORD expression was observed in the serum of some HCC patients after SR. Those patients demonstrated a shorter RFS rate as compared to patients with low serum SORD. To conclude, SORD might be a remarkable biomarker for HCC in the late stage, which shows more cancer aggressiveness that might be related to drug resistance in HCC. Further studies are required to investigate its direct relation to drug resistance and its use in the early detection of HCC.

Our proteomics results demonstrated that UDP-glucose-6-dehydrogenase (UGDH) was significantly increased in resistant Hep3B cells compared to parental phenotype (Figure 5B). UGDH is a rate-limiting enzyme in the glucuronic acid pathway, which is involved in xenobiotics metabolism and elimination (Supplementary material: Summary Table S2). UGDH catalyzes the production of UDP-glucuronic acid (UDP-GlcUA) from UDP-glucose (UDP-Glc). UDP-GlcUA is metabolized as glycosaminoglycans (GAGs) precursor-like hyaluronic acid and in glucuronidation reaction, conjugation with xenobiotic functional groups via UDP-glucuronosyltransferases, which increases the polarity of the compound for faster elimination [91]. The effect of UGDH in promoting metastasis in colorectal carcinoma through GAG production has been previously reported [92]. UGDH knockout attenuates the migration of breast cancer cells in vivo [93]. In the context of UGDH role in chemoresistance, sensitivity of HCC to sorafenib might be improved by combining UGDH depletion with sorafenib. Sorafenib activates the glucuronic acid metabolism and elevates the UGDH in HCC patients [94]. Combining UGHD depletion and sorafenib can reduce the levels of UGDH and consequently inhibits the unfolded protein response (UPR), which participates in controlling cell survival and apoptosis [94]. Thus, UGDH elevated levels in resistant HCC cells might be a promising target in chemotherapeutic drug resistance. 

Calpain small subunit 1 (CAPNS1) significantly decreased in resistant Hep3B cells compared to its parental phenotype (Figure 5B). CAPNS1 is a cysteine proteinase that functions in catalyzing the limited proteolysis of various substrates in many physiological processes, including cellular signaling, apoptosis, cytoskeletal remodeling and cell survival [95]. It has been shown that resistant gastric cancer cells demonstrate upregulated levels of miR-99a and miR-491 gene expression regulators, which are highly associated with drug resistance [96] and CAPNS1 gene downregulation. Inhibition of miR-99a and miR-491 leads to overexpression of CAPNS1, which augments the sensitivity to cisplatin in the resistant cells [97]. Therefore, targeting CAPNS1 in resistant cells could be valuable in chemo-sensitizing approaches in resistant HCC cells. 

### 3.3. Top Significantly Enriched Gene Ontology Biological Process Terms Indicate Dysregulation in Processes Linked to Energy Production, Anabolism and Cancer Cell Survival and Growth

Gene ontology enrichment analysis of the significantly differentially abundant proteins (*p* value > 0.05) between the resistant and parental Hep3B cells demonstrated their significant participation in many processes that support the cancer cell survival (Figure 6). Most of these processes are mainly related to energy generation and maintenance of homeostasis such as the NADP biosynthetic process, protein targeting to lysosome involved in chaperone-mediated autophagy, chaperone-mediated autophagy, farnesyl diphosphate biosynthesis, protein unfolding, and protein insertion into mitochondrial outer membrane (Figure 6). It is well-known that tumor cells protect themselves from apoptosis and DNA damage by maintaining the redox homeostasis and overcoming the oxidative stress produced by ROS overexpression through developing complicated antioxidant defense mechanisms that utilize various antioxidant enzymes such as glutathione reductase [98]. These antioxidant enzymes are nicotinamide adenine dinucleotide phosphate (NADPH)-dependent [44], which maintain the reduction potential in several anabolic reactions [99]. These anabolic reactions might aid in cell survival.

Protein targeting to lysosome involved in chaperone-mediated autophagy and chaperone-mediated autophagy (CMA) were among the top significantly enriched GO terms (Figure 6). CMA is an oncogenic process that supports cancer cell survival and growth via maintaining the energy supply and cellular homeostasis [40]. Furthermore, protein unfolding and protein insertion into the mitochondrial outer membrane were significantly enriched in our GO enrichment analysis (Figure 6). Tumor cells, in consequence of cellular stresses, such as hypoxia and nutrient deprivation, exhibit inefficient protein folding in the endoplasmic reticulum leading to the accumulation of unfolded proteins, which cause ER stress. Subsequently, unfolded proteins will trigger the unfolded protein response (UPR) to restore the ER homeostasis and maintain cancer survival [41]. Interestingly, to maintain efficient cellular functions and biosynthesis, protein insertion into the outer membrane supports cell viability [100]. Cancer reprograms mitochondrial functions to drive aerobic glycolysis and boost ATP production [101]. Low ATP levels during glycolysis trigger the mitochondria to upregulate import proteins to increase the ATP generation [39]. It is worth noting that “telomerase holoenzyme complex assembly” and “regulation of mRNA stability involved in response to stress” were significantly enriched in our GOEA results (Figure 6). Telomerase in cancer is critical to maintaining the telomeric length that is vital for cancer development and survival [36]. It has been found that, mortalin; stress-inducible molecular chaperone, can activate telomerase functions enhancing tumor progression, survival and sorafenib resistance [102,103]. Moreover, regulation of mRNA stability is important for gene expression and sustaining mRNA’s availability for translation [43].

### 3.4. Multi-Omics Joint Pathway Enrichment Analysis Revealed Unique Pathways That Might be Druggable Targets in the Resistant HCC

In our joint pathway enrichment analysis, the spectrum of pathways was enriched, and the molecular signatures of each were related to cancer. A subset of pathways was significantly associated with protein biosynthesis, misfolding and hemostasis (Figure 7). Unique pathways in terms of protein production involves ribosome pathway and aminoacyl-tRNA biosynthesis. Ribosome aids in upsurging protein synthesis to support cancer growth [104]. In a recent published article, it was stated that ribosomal biogenesis up-regulation and transcriptome alteration aid in tamoxifen resistance development via translation profile modulation of c-MYC, which is linked to tamoxifen resistance [105]. Aminoacyl-tRNA biosynthesis plays a key role in transferring amino acids to tRNA in protein translation and protein biogenesis [106]. The other subset of pathways that were significantly enriched and related to transcription includes spliceosome and PPAR signaling pathway (Figure 7). The spliceosome is an RNA-protein complex that aids in introns removal and splicing from pre-mRNA in the nucleus [107]. Various factors can alter splicing in cancer cells like spliceosome mutation, deregulation of transcription, post-transitional modifications, long non-coding RNAs (lncRNAs) and microRNA. Consequently, spliceosome function and localization are altered, leading to cell signaling alteration [108]. Additionally, splicing events in cancer-related genes generate mRNA variants and this encode proteins with differential functions and structure that affect the cancer response to treatment and promote resistance [109]. Moreover, PPARs are nuclear hormone receptors regulating gene expression, cell proliferation, energy hemostasis and apoptosis [110]. As discussed in Section 3.1, inhibiting (PPARδ) will affect the glutamine metabolism in sorafenib-resistant HCC and combat sorafenib resistance [55].

Added to the enriched amino acid and nucleotide metabolic pathways identified in the metabolomic analysis, the pyruvate pathway was significantly enriched in the joint pathway enrichment analysis (Figure 7). In cancer cells, the Warburg Effect boosts the glucose consumption to undergo glycolysis or gluconeogenesis, which was highly enriched in our joint pathway analysis (Figure 7). This may cause pyruvate production and conversion to lactate. These pathways support the growth and survival of cancer cells through energy production and driving anabolic pathways [111]. In addition, pyruvate kinase (PKM2) is a pyruvate flux gatekeeper [112] that playes an important role in drug-resistance in many types of cancer. PKM2 expression was demonstrated to be upregulated in enzalutamide-resistant prostate cancer [113]. Additionally, fatty acid degradation, central carbon metabolism in cancer, protein digestion and absorption, and TCA cycle were significantly enriched in the joint pathway enrichment analysis (Figure 7), which are key pathways in energy production required for the survival of cancer cells [47]. Synthesis and degradation of ketone bodies were also significantly enriched pathways in our GOEA (Figure 7). During nutrient deprivation in HCC, metabolic pathways, such as 3-oxoacid CoA-transferase 1, are upregulated to enhance the energy supply through ketone body consumption [114]. Additionally, the ATP production linked to ketone body may reduce the phosphorylation of AMP-activated protein kinase (AMPK) as well as autophagy [114]. 

The joint pathway analysis of the significant metabolomics and proteomics profiles of resistant Hep3B cells compared to parental phenotype revealed various pathways that aids in understanding the mechanism of drug resistance, and whose targeting could improve the therapeutic outcomes and assist future studies into the pathophysiological processes in HCC. 

## 4. Materials and Methods

### 4.1. Reagents

Methanol (≥99.9%), acetonitrile (ACN) and deionized water, as well as LC-MS CHROMASOLV, were purchased from Honeywell (Wunstorfer, Strasse, Seelze, Germany). Trifluoroacetic acid (TFA) and formic acid (FA) were purchased from Fisher Scientific (Loughborough, UK). Hydrochloric acid (HCl) (37%) was purchased from VWR chemicals (France). C18 columns, lysis buffer, Pierce trypsin protease, lysyl-endopeptidase LysC and Pierce protease inhibitor tablets were obtained from Thermo Scientific (Rockford, IL, USA). Bradford’s reagent and bovine serum albumin were bought from Sigma-Aldrich (St. Louis, MO, USA). Sorafenib was obtained from BioVision (Milpitas, CA, USA #BAY 43-9006). 

### 4.2. Cell Lines and Culture Conditions

The Hep3B cell line was selected as the in vitro model for this study because it is one of the most widely used and well-characterized liver cancer cell lines. It is frequently used as an experimental model and is used as a cellular reference model in pharmaceutical studies that aim to develop new drugs and gain insights into drug metabolism, including knowledge of involved enzymes and the drug’s inhibition or induction potential [115]. The Hep3B cell line used in this study was purchased from Sigma-Aldrich (St. Louis, MO, USA). Sorafenib-resistant Hep3B cells were developed from Hep3B cell lines as described in Section 2.3. One million Hep3B cells were seeded in T75 flasks and cultured in Dulbecco’s Modified Eagle Medium (DMEM) as monolayers. The medium was supplemented with 10% fetal bovine serum (FBS) (Sigma Aldrich, St. Louis, #F2442-500ML) and 1% penicillin/streptomycin (Sigma Aldrich, St. Louis, #P4333). Cultures were then incubated in 5% CO_2_ humidified atmosphere at 37 °C for 3–4 days until 70–80% confluency was reached. Aseptic techniques were applied to avoid potential contamination, and the confluency and contamination were checked routinely. 

### 4.3. Generation of Resistant Hep3B Cell Line

Resistant Hep3B cells were developed in clinically relevant models [116], as previously reported [117]. In T-75 flasks, Hep3B cells were seeded overnight and incubated at 37 °C. The cells were then treated with sorafenib at the 10% inhibitory concentration (IC10) (0.4 µM) of sorafenib (Biovision, Milpitas, CA, USA #BAY 43-9006) to mimic the clinically consumed low dose of chemotherapeutic drugs by a cancer patient who is then exposed to escalating doses over time [116]. To develop resistance, the survived cells were transplanted to a new flask and then treated with sorafenib at escalating concentrations over six months. Then, sorafenib at concentration IC10 (0.4 µM) was persistently retained in the culture media to ensure and maintain resistance. MTT cell viability assay was performed each month to validate the resistance behavior of the cells.

### 4.4. MTT Cell Viability Assay

The MTT cell viability assay was carried out in triplicates as described previously [118]. Briefly, parental and resistant Hep3B cells were seeded in 96-well plates (4 × 103 cells per well) for 24 h. To determine the sorafenib related half-maximal inhibitory concentration (IC50), each cell line was treated with different concentrations of sorafenib (Sigma-Aldrich, Steinheim, Germany) for 48 h. Afterwards, 200 μL of MTT solution (5 mg/mL) (Sigma-Aldrich, Germany) was applied to replace the media, and the cultures were incubated at 37 °C for 2 h. Then, a 200 μL volume of dimethyl sulfoxide (DMSO) was added to dissolve the formed formazan crystals. Lastly, Varioskan Flash (Thermo Fisher Scientific, Waltham, MA, USA) was utilized to determine the absorbance at 570 nm.

### 4.5. Metabolite Extraction

For metabolomics, three biological replicates were used. An equal number of cells were utilized for each sample. The cells were centrifuged separately at 15000 rpm for 10 min to separate the cells and cell-free supernatants. The supernatants were discarded, and to each cell pellet (~3 × 10^6^), 1 mL of 0.1% formic acid in methanol was added, followed by vertexing for 2 min × 4 and interrupted by 15 min incubation on ice. This was followed by sonication using a COPLEY probe-sonicator (QSONICA SONICATOR, USA) for 30 s with 30% amplifier in an ice bath. Subsequently, the cellular debris of each sample was collected by centrifugation at 15,000 rpm for 10 min at 4 °C. The supernatants were separated and dried at 37 ± 1 °C using EZ-2 Plus (GeneVac, Ipswich, UK). Finally, 200 µL of 0.1% formic acid in water was used to resuspend the dried samples, which were then vortexed for 2 min, filtered by a 0.45 µm hydrophilic nylon syringe filter and transferred to 200 μL (micro-inserts) in LC vials. The quality control (QC) sample was prepared by pooling the same volume (10 μL) of each sample, and all samples were placed in the autosampler at a temperature set at 4 °C and analyzed with UHPLC-QTOF-MS.

### 4.6. Protein Extraction and Quantification

For proteomics, three biological replicates were used. In protein extraction, each cell pellet (~3 × 10^6^) was lysed by 400 µL lysis buffer (Catalog number: FNN0011, Thermo Fisher Scientific, USA) made by dissolving one tablet of protease inhibitors in 10 mL lysis buffer (1× protease inhibitors:10 mL Lysis buffer) for 10 min. This was followed by sonication at 30% amplifier for 30 sec and then centrifuged at 15,000 rpm for 5 min at 4 °C. The supernatants were then collected in 1.5 mL microcentrifuge tubes. To precipitate the cell proteins, 400 µL methanol and 300 µL chloroform were added to each sample and vortexed for 1 min. Next, all samples were centrifuged at 13,000 rpm for 5 min at 4 °C to generate a white protein disk interface in biphasic solution. The supernatant was thrown out, while the bottom phase was vortexed after the addition of 300 µL methanol and then centrifuged at 13,000 rpm for 1 min at 4 °C. The formed supernatant was again discarded, and the resultant protein pellet was air-dried and then resuspended in denaturation buffer (2M thiourea and 6M urea in 10mM Tris buffer at pH 8). 

### 4.7. Bradford Assay

To quantify the protein in all samples, a modified Bradford assay was performed as previously described [119]. The modified Bradford assay was chosen since the urea in the denaturation buffer interferes with the standard Bradford. In this case, acidification of the sample buffer is required to obtain stable standard curves [119]. Briefly, the standard curve of bovine serum albumin (BSA) was prepared using diluted BSA stock (10mg/mL) with deionized water in (5:100) dilution. Aliquots in volumes of 0, 0.5, 1, 2, 4 and 8 µL were selected, and all were performed in triplicate. For each sample, two volumes were selected (1 and 2 µL) and performed in duplicate to avoid wasting the samples. First, a volume of 90 µL of HCl 1:8 (HCl 0.1N: deionized water) was applied to 96-well plate for the standard and samples. Then, the selected volumes of the standard and the samples were added and diluted with deionized water to reach a final volume of 9 µL followed by agitation for 5 min. Then, 150 µL Bradford’s reagent: deionized water (1:2) was added and agitated for 5 min. Finally, the absorbance was measured at 570 nm using Varioskan Flash (Thermo Fisher Scientific, Waltham, MA, USA).

### 4.8. In-Solution Protein Digestion and Peptide Desalting

In-solution protein digestion was performed as previously described [120]. Briefly, protein samples (100 µg) were reduced using dithiothreitol (DTT) at 1 mM followed by incubation for 1 h with gentle agitation at 100 rpm under room temperature (RT). Afterwards, the samples were alkylated using iodoacetamide (IAA) at 5.5 mM and incubated in the dark for 1 h at 100 rpm and RT. After each step, the pH was adjusted, if necessary, to 8.0. Then, 1 µg of Lysyl Endopeptidase LysC was added (1:100, *w*/*w*), and the samples were incubated for 3 h with 100rpm at RT. Subsequently, the samples were diluted four times using 20 mM ammonium bicarbonate and then digested with 1 µg trypsin (1:100 ratio) and incubated overnight at 100 rpm and RT.

For desalting, all samples were dried and resuspended using 1% trifluoroacetic acid (TFA) and filtered using commercial C18 STAGE (stop and go extraction) tips. C18 membranes were first activated by repeatedly aspirating and dispensing 100 µL 50% ACN followed by equilibration using 100 µL of 0.1% TFA. The digested protein samples were loaded by repetitively aspirating and dispensing the samples ten times. The columns were then rinsed with 0.1% TFA in 5% ACN. The peptides were then eluted into LC vials through careful aspiration and dispensing of 100 µL of 0.1% formic acid (FA) in 60% ACN. The resulted desalted peptides were then dried using EZ-2 Plus (GeneVac, Ipswich, UK) and resuspended in 0.1% FA in 2% ACN prior to LC-MS/MS analysis.

### 4.9. Ultra-High-Performance Liquid Chromatography-Tandem Mass Spectrometry (UHPLC-MS/MS)

The Elute UHPL and Q-TOF Mass Spectrometer (Bruker, Bremen, Germany) were utilized for metabolite and peptide detection. The Elute HPG 1300 pumps, Elute Autosampler (Bruker, Bremen, Germany) and Hamilton^®^ Intensity Solo 2 C18 column (100 mm × 2.1 mm, 1.8 m beads) were employed using reversed-phase chromatography. Solvents used for separation were 0.1% FA in LC grade water (solvent A) and 0.1% FA in ACN (solvent B). 

Each metabolite and protein extract were analyzed in duplicate. For metabolomics analysis, the column was kept at 35 °C, and each sample was injected twice with an injection volume of 10 µL. Sample elution was performed in 30 min gradient starting with 1% ACN for 2 min and then ramped to 99% ACN within 15 min. After that, 99% ACN was kept for 3 min, and then the re-equilibration to 1% ACN was done for 10 min. The flow rate was 0.25 mL/min for 20 min and then 0.35 mL/min for 8.3 min and then the flow rate set at 0.25 mL/min for 1.7 min.

For MS2 acquisition in metabolomics analysis, the collision energy was fluctuated between 100–250% of 20 eV and end plate offset of 500 V. The acquisition was in two sections: auto MS scan for the calibrant sodium formate in 0–0.3 min, and auto MS/MS for fragmentation, in 0.3 to 30 min. Positive mode at 12 Hz was performed in both acquisition sections. The scan range was 20 to 1300 *m*/*z*, the precursor ion’s width of ±0.5, the precursors number of 3, the cycle time of 0.5 s, and the threshold of 400 counts. After three spectra, active exclusion was performed and released after 0.2 min.

For proteomics analysis, the column was kept at 32 °C and an amount of 10 µg of peptide in each sample was injected twice. Sample elution was achieved through a 110-min gradient starting with 5% ACN for 5 min, then gradually increased to 35% ACN during 85 min, until it reached 95% ACN over 5 min. The back pressure values were < 350 bar during the separation, and the flow rate was steady at 300 μL/min.

A Q-TOF (Bruker, Bremen, Germany) with an Apollo II electrospray ionization (ESI) source was utilized for the MS analysis with the following parameters. The nebulizer pressure was 2.2 bar, the drying gas flow rate was 10 L/min, the drying temperature was 220 °C and the capillary voltage was 4500 V. The scan range for proteomics was 150–2200 *m*/*z* and peptides were analyzed in auto-MS/MS mode, with 100 eV collision energy, a target intensity of 10,000 counts per second (cps), a minimum relative intensity threshold of 500 counts per thousand, a fixed cycle length of 3 s and an analyte charge of 2 ≥ x ≥ 5. In the first 0.3 min of each LC-MS/MS run, the external calibrant, sodium formate, was injected. Mass calibration was done prior to analysis according to the manufacturer’s recommendations using external mass calibration (10 mM sodium formate calibrant solution). The performance of the column and the mass spectrometer was tested using a test mixture of (TRX-2101/RT-28-calibrants for Bruker T-ReX LC-QTOF solution from Nova Medical Testing Inc.) to check the performance of reversed-phase liquid chromatography (RPLC) separation and perform multipoint retention time calibration and (TRX-3112-R/MS certified human serum for Bruker T-ReX LC-QTOF solution from Nova Medical Testing Inc.) to check the performance of sample preparation protocols, as well as LC-MS instruments. This product was prepared from pooled human blood.

### 4.10. Data Analysis and Statistical Approach

#### 4.10.1. Metabolomics Data Processing

For metabolomic analysis, MetaboScape^®^ 4.0 program was employed (Bruker Daltonics, Billerica, MA, USA). In the T-ReX 2D/3D workflow, to identify the molecular features, a minimum peak length was set to 7 spectra and the minimum intensity threshold was 1000 counts for peaks detection. The peak area was employed for quantification and the injected external calibrant in the interval of 0–0.3 min was used to recalibrate the mass spectra. The selected mass to charge ratio (*m*/*z*) and retention time for scanning were in the ranges of 50–1000 *m*/*z* and 0.3–25 min, respectively. The auto MS/MS scanning method was set to average. Features found at least in 3 of the 12 samples were the only ones taken into further consideration. MetaboScape^®^ was used to detect the metabolites by matching them to the human metabolome database (HMDB) in two ways: matching with MS2 spectra and retention time. Where multiple features’ matched to a given database entry, the annotation quality score (AQ score) was used to select only the best matching feature. A greater the AQ score denoted the best overall match by considering which features best fits the combined MS/MS, precursor *m*/*z* values, retention time and isotopic pattern scores. 

The resulting metabolite quantitation data were scaled to unit variance and mean-centered, and principal component analysis (PCA) and sparse partial least squares-discriminant analysis (sPLS-DA) were applied, using MetaboAnalyst, to visualize the metabolomic data. Two-tailed independent students T-test utilising MetaboScape^®^ were used to identify the significant differences in metabolite abundance between parental and resistant Hep3B cells. Features with a *p* value < 0.05 and a log2 (fold-change) more than two were considered statistically significant. MetaboAnalyst was utilized to obtain the volcano plot and the heatmap with hierarchical clustering (*p* value < 0.05 and log2(fold-change) > 2) and the pathway enrichment analyses for the significant metabolites sets (*p* value < 0.05) through over-representation analysis (ORA) available at https://www.metaboanalyst.ca. ORA examines if a certain set of metabolites is represented more than anticipated by chance within the uploaded metabolite list through hypergeometric test.

#### 4.10.2. Proteomics Data Processing

To identify the proteins and peptides, the raw data were processed by way of MaxQuant 1.6.17.0 [121] using the Uniprot proteome for Homo sapiens (Proteome ID: UP000005640, 79,052 entries, 14 April 2022) and the Andromeda search engine. Default parameters were applied in the MS/MS database search, including methionine oxidation and acetylation of protein N-termini as variable modifications and carbamidomethylation of cysteine residues as a fixed modification. Peptide spectral matching (PSMs) was filtered with a 20-ppm precursor mass tolerance and 1% false discovery rate (FDR). For label-free quantification (LFQ) [122], the MaxLFQ algorithm was used. In the in-silico digestion, the default trypsin/P enzymatic cleavage rule was utilized. To ensure a reliable label-free quantification, the protein groups were identified with at least two peptides and were selected for further downstream analysis. Perseus software 2.0.5.0 was used for further data processing and statistical analysis. The potential contaminant proteins, along with the proteins only identified by site and reverse proteins, were filtered out from the data. LFQ-values were transformed into log2(x). Proteins were annotated, and those with 70% valid values in total were preserved for analysis. Subsequently, imputation was done by replacing the missing values from a normal distribution calculated separately for each sample with a downshift of 1.8 and width of 0.3. To visualize the data after imputation, PCA was performed. Two-tailed independent student *t*-test was utilized to identify the significantly expressed proteins in the parental and resistant Hep3B cells. Multiple testing correction was done using the Benjamini-Hochberg procedure. Proteins were considered differentially expressed if the adjusted *p* value was < 0.05 and log2 fold change was more than 1. A volcano plot was used to visualize the deregulated proteins. Hierarchical clustering and a heatmap were applied to the differentially expressed proteins to assess their expression in each group and to visualize group clustering. Significantly, dysregulated proteins with an adjusted *p* value < 0.5 were analyzed for enrichment using the GOEA using String software version 11.5 to visualize the biological process gene ontology (GO) terms.

#### 4.10.3. Joint Pathway Analysis

Metaboanalyst was utilized for the joint pathway analysis. The entry IDs of the 212 significant proteins (*p* value < 0.5) and names of the 39 significant metabolites (*p* value < 0.05) were applied in the joint pathway analysis tool to visualize the enriched pathways.

## 5. Conclusions

Our findings revealed that sorafenib-resistant Hep3B cells exhibited a significant alteration in amino acid and nucleotide metabolism, energy production and other pathways linked to cancer aggressiveness, migration, proliferation and drug-resistance. D-alanine, o-tyrosine and PC (16:0/16:0) were among the significantly altered metabolites we proposed as potential biomarkers for drug resistance in HCC. Promising potential proteomics biomarkers for drug resistance in HCC include UCHL1, mitochondrial SOD2, UGDH, SORD and CAPNS1. Unique pathways were identified in the joint pathway enrichment analysis, including the antifolate resistance pathway that might contribute to MDR in HCC and other extremely important pathways that maintain cancer cell survival, growth and proliferation. Our results shed light on the molecular basis of chemotherapeutic drug resistance, cancer initiation and progression and identifying potential diagnostic and/or prognostic biomarkers for HCC.

## Figures and Tables

**Table 1:** Significant deregulated metabolites in resistant Hep3B cells in comparison to parental Hep3B cells.

Metabolite Name	*p*-Value	Fold Change
Uridine 5′-monophosphate	0.000002	14.729
ADP	0.000001	13.186
Adenosine monophosphate	0.000778	12.894
PC (16:0/16:0)	0.000219	11.971
Guanosine monophosphate	0.000521	10.978
Adenine	0.000012	10.673
Cyclic AMP	0.000006	8.304
Cytosine	0.000071	6.264
Urocanic acid	0.037089	5.207
Succinic acid	0.001392	5.133
Glycerophosphocholine	0.000014	5.088
Deoxyguanosine	0.000114	4.233
Creatine	0.000253	3.801
Phenylpropanolamine	0.006535	3.672
Guaifenesin	0.043493	2.978
Picolinic acid	0.000545	2.911
Pyridine	0.007763	2.874
D-Alanine	0.019774	2.688
Niacinamide	0.012453	2.55
L-Tryptophan	0.015553	2.39
L-Proline	0.007556	2.372
Uridine diphosphate-N-acetylglucosamine	0.013429	2.253
Pyroglutamic acid	0.033094	2.238
Cinnamic acid	0.037616	2.159
o-Tyrosine	0.049019	2.098
L-Phenylalanine	0.039898	2.052
L-Arginine	0.04468	−21.951

**Table 2:** Gene ID refer to proteins significantly deregulated in resistant Hep3B cells in comparison to Parental Hep3B cells.

Uniprot ID	Adjusted *p* Value	Effect Size	Significance
Q9C0H2	0.000315988	−2.831839879	Decreased
P07148	4.39 × 10^−6^	−2.285889943	Decreased
Q12769	0.009665038	−2.158726692	Decreased
O95573	0.000719872	−1.838945548	Decreased
P49591	0.001699466	−1.737114747	Decreased
Q9BWD1	3.18 × 10^−10^	−1.703698476	Decreased
P13473	0.009289266	−1.515532017	Decreased
P04632	0.005943185	−1.197470983	Decreased
Q01581	0.001058751	−1.166633765	Decreased
Q53GQ0	6.19 × 10^−6^	−1.081565698	Decreased
P49327	1.72 × 10^−10^	−1.074158986	Decreased
P27487	0.000121063	−1.02650706	Decreased
P17174	1.74 × 10^−6^	−1.015660763	Decreased
P04179	7.79 × 10^−5^	1.049084345	Increased
Q9P035	0.003254458	1.252471606	Increased
Q00796	0.000258132	1.474272092	Increased
O60701	4.02 × 10^−13^	2.068751653	Increased
P09936	2.41 × 10^−10^	2.986876806	Increased

## Data Availability

The metabolomics data have been deposited in the Metabolomics Workbench repository at 10.21228/M8S71T with data ID ST002273. The proteomics data are uploaded to PRIDE and the reference number is 1-20220915-32336.

## Supplementary Materials

The following supporting information can be downloaded at: https://www.mdpi.com/article/10.3390/ijms231911975/s1.
