# Supplementary material for: Multi-Omics Analysis Revealed a Significant Alteration of Critical Metabolic Pathways Due to Sorafenib-Resistance in Hep3B Cell Lines
Source: Int J Mol Sci. 2022 Oct 9;23(19):11975. doi: 10.3390/ijms231911975 (PMC9569810; doi:10.3390/ijms231911975)
Supplement: Supplementary file 1 [file ijms-23-11975-s001.zip › Figure S1.edited.pdf]

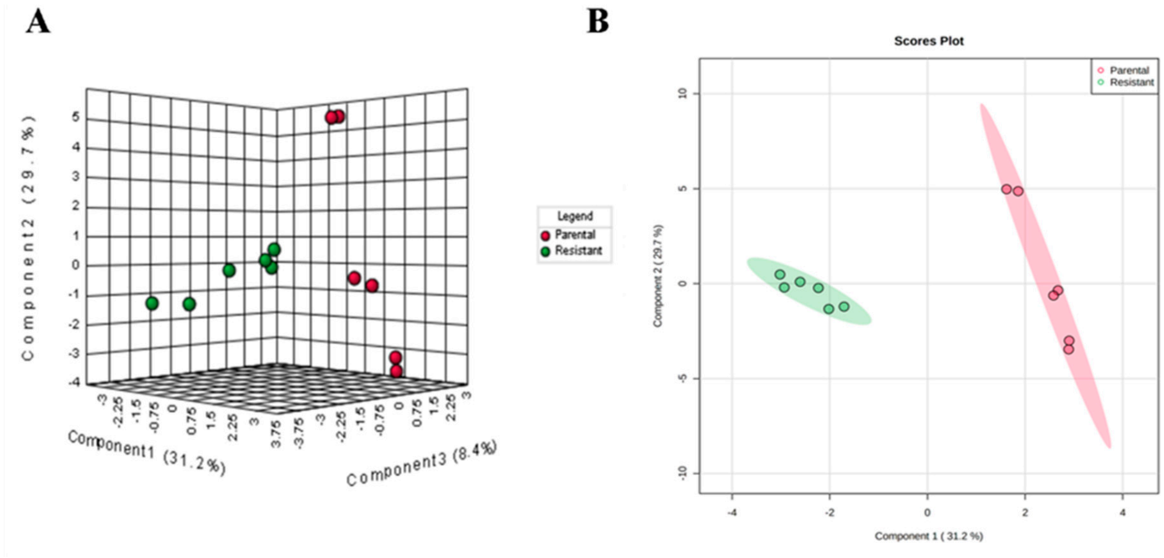


**Figure S1:** The sparse partial least squares-discriminant analysis (sPLS-Da) of parental and resistant Hep3B cells. (A) 3-D scores plot between selected components: (B) 2-D scores plot between selected components.
