# Supplementary material for: Multi-Omics Analysis Revealed a Significant Alteration of Critical Metabolic Pathways Due to Sorafenib-Resistance in Hep3B Cell Lines
Source: Int J Mol Sci. 2022 Oct 9;23(19):11975. doi: 10.3390/ijms231911975 (PMC9569810; doi:10.3390/ijms231911975)
Supplement: Supplementary file 1 [file ijms-23-11975-s001.zip › Tables.pdf]

Summary Table S1. Summary of the significant **decreased proteins levels** in resistant Hep3B cells vs. Parental Hep3B cells.

Protein UniProt ID	Protein description	Abundance levels	GO – Biological process * (source Uniprot)	Possible Association with Liver cancer or any other cancer
Q9C0H2	Protein tweety homolog 3	Decrease	chloride transport, ion transmembrane transport	TTYH3 overexpression supports cell migration, proliferation, and invasion in cholangiocarcinoma (CCA), Overexpression of TTYH3 in ovarian cancer suggested worse prognosis and clinical outcomes,
P07148	Fatty acid-binding protein, liver	Decrease	cellular response to hydrogen peroxide, negative regulation of apoptotic process, negative regulation of cysteine-type endopeptidase activity involved in apoptotic process	L-FABP has a significant function in oxidative stress, L-FABP occurs at the adenoma stage of colorectal tumour development and indicates that L-FABP is a marker of colorectal cancer differentiation
Q12769	Nuclear pore complex protein Nup160	Decrease	mRNA export from nucleus, nucleocytoplasmic transport, protein transport	Inhibition of nuclear pore complex stimulates cancer cell death
O95573	Fatty acid CoA ligase Acsl3	Decrease	fatty acid metabolic process, very-low-density lipoprotein particle assembly, positive regulation of phosphatidylcholine biosynthetic process	IL-6 stimulates miR-603 expression that inhibits FABP1 expression, encourages the protein synthesis, lipid metabolism, elevated cellular oxidative stress, that causes metastasis of HCC, Suppression of Acsl3 triggers death in NSCLC cell lines.

* Source: UniProt, available at **https://www.uniprot.org/**

Cont. Summary Table S2.

Protein UniProt ID	Protein description	Abundance levels	GO – Biological process *	Possible Association with Liver cancer or any other cancer
P49591	Serine--tRNA ligase, cytoplasmic	Decrease	negative regulation of angiogenesis, negative regulation of transcription by RNA polymerase II,	Serine biosynthesis pathway is crucial in BRAF inhibitor resistance of some cancer cells.
Q9BWD1	Acetyl-CoA acetyltransferase, cytosolic	Decrease	fatty acid beta-oxidation, lipid metabolic process	ACAT2 stimulates cell proliferation in colorectal cancer, HADHA and ACAT2 down-expression were associated with poorer prognosis in clear cell renal cell carcinoma.
P13473	Lysosome-associated membrane glycoprotein 2	Decrease	cellular response to starvation, autophagosome maturation, chaperone-mediated autophagy, protein targeting to lysosome involved in chaperone-mediated autophagy	LAMP2 overexpression indicates poor prognosis esophageal squamous cell carcinoma patients
P04632	Calpain small subunit 1	Decrease	positive regulation of cell population proliferation, regulation of macroautophagy	Cisplatin resistance in human gastric cancer cells can be regulated by MiR-99a and MiR-491 through targeting CAPNS1
Q01581	Hydroxymethylglutaryl-CoA synthase, cytoplasmic	Decrease	acetyl-CoA metabolic process, farnesyl diphosphate biosynthetic process, mevalonate pathway, lipid metabolic process	-

* Source: UniProt, available at **https://www.uniprot.org/**

Cont. Summary Table S3.

Protein UniProt ID	Protein description	Abundance levels	GO – Biological process *	Possible Association with Liver cancer or any other cancer
Q53GQ0	Very-long-chain 3-oxoacyl-CoA reductase	Decrease	extracellular matrix organisation, positive regulation of cell-substrate adhesion, estrogen biosynthetic process	-
P49327	Fatty acid synthase	Decrease	cellular response to interleukin 4, inflammatory response, neutrophil differentiation, monocyte differentiation	Fatty acid synthase inhibition leads to osteosarcoma cell invasion and migration suppression by PI3K/Akt signalling pathway downregulation
P27487	Dipeptidyl peptidase 4	Decrease	cell adhesion, endothelial cell migration, positive regulation of cell population proliferation	Dipeptidyl peptidase 4 inhibitors activate lymphocyte chemotaxis in mice reduce and causes hepatocellular carcinoma suppression.
P17174	Aspartate aminotransferase, cytoplasmic	Decrease	Notch signalling pathway, response to glucocorticoid, glutamate catabolic process to aspartate	Resistance to the oxamate cytostatic effects in breast cancer resulted from over. expression of Aspartate aminotransferase (AAT).

* Source: UniProt, available at **https://www.uniprot.org/**

Summary Table S2. Summary of the significant **increased proteins levels** in resistant Hep3B cells vs. Parental phenotype

Protein UniProt ID	Protein description	Abundance levels	GO – Biological process *	Possible Association with Liver cancer or any other cancer
P04179	Superoxide dismutase [Mn], mitochondrial	Increase	hydrogen peroxide biosynthetic process, intrinsic apoptotic signalling pathway in response to DNA damage, intrinsic apoptotic signalling pathway in response to oxidative stress, negative regulation of cell population proliferation, positive regulation of cell migration, positive regulation of nitric oxide biosynthetic process	Mn-SOD overexpressed in ovarian cancer in response to oxidative stress in cancer cells. Endometriosis- associated ovarian cancer (EAOC) demonstrated high SOD2 expression which might be prognostic biomarker for its poor prognosis.
Q9P035	Very-long-chain (3R)-3-hydroxyacyl-CoA dehydratase 3	Increase	positive regulation by virus of viral protein levels in host cell, positive regulation of viral genome replication, very long-chain fatty acid biosynthetic process	Very-long-chain (3R)-3-hydroxyacyl-CoA dehydratase 3 is linked with I-kappaB kinase/ NF-kappaB signalling pathways that might contribute to Male breast cancer tumorigenesis.
Q00796	Sorbitol dehydrogenase	Increase	glucose metabolic process, response to xenobiotic stimulus, sorbitol catabolic process	Serum SORD along with serum AFP levels is considered predictive factor for HCC developed after resection, SORD is expressed in proliferating adenomas cells associated with high fructose-driven metabolic processes.

* Source: UniProt, available at **https://www.uniprot.org/**

Cont. Summary Table S2.

Protein UniProt ID	Protein description	Abundance levels	GO – Biological process *	Possible Association with Liver cancer or any other cancer
O60701	UDP-glucose 6-dehydrogenase	Increase	carbohydrate metabolic process, heparan sulphate proteoglycan biosynthetic process, gastrulation with mouth forming second, glycosaminoglycan biosynthetic process	UGDH is necessary for cell migration induced by krÜppel-like factor 4 (KLF4) and cell motility through the production of glycosaminoglycans (GAG) in glioblastoma cells.
P09936	Ubiquitin carboxyl-terminal hydrolase isozyme L1	Increase	negative regulation of MAP kinase activity, cellular response to xenobiotic stimulus, cell population proliferation, regulation of macroautophagy, protein deubiquitination	UCH-L1 is speculated to be crucial in regulating tumour invasion vi stimulation of Akt-mediated pathway in non-small lung cancer cell line H157 , UCH-L1 in many tumour types has impact in the regulation of metastasis and cell growth, In lung adenocarcinoma UCHL1 might be a potential target and shows a potential oncogenic role, Ubiquitin carboxyl terminal hydrolase-l1 overexpression assisted multi-drug resistance, metastasis and invasion in breast cancer.

* Source: UniProt, available at **https://www.uniprot.org/**
